# Barriers and facilitators to the adoption of physical activity policies in elementary schools from the perspective of principals: An application of the consolidated framework for implementation research–A cross-sectional study

**DOI:** 10.3389/fpubh.2023.935292

**Published:** 2023-02-22

**Authors:** Janine Wendt, Daniel A. Scheller, Marion Flechtner-Mors, Biljana Meshkovska, Aleksandra Luszczynska, Nanna Lien, Sarah Forberger, Anna Banik, Karolina Lobczowska, Jürgen M. Steinacker

**Affiliations:** ^1^Division of Sports and Rehabilitation Medicine, Department of Internal Medicine, University Hospital Ulm, Ulm, Germany; ^2^Department of Nutrition, Faculty of Medicine, Institute of Basic Medical Sciences, University of Oslo, Oslo, Norway; ^3^Department of Psychology, SWPS University of Social Sciences and Humanities, Wroclaw, Poland; ^4^Melbourne School of Psychological Sciences, Melbourne Centre for Behavior Change, University of Melbourne, Melbourne, VIC, Australia; ^5^Department of Prevention and Evaluation, Leibniz Institute for Prevention Research and Epidemiology–BIPS, Bremen, Germany

**Keywords:** implementation outcome, implementation determinants, health promotion, schoolchildren, implementation framework

## Abstract

**Background:**

Studies have shown that policies to promote physical activity in schools can have a positive impact on children's physical activity behavior. However, a large research gap exists as to what determinants may influence the adoption of such policies. Applying the Consolidated Framework for Implementation Research (CFIR), we investigated barriers and facilitators to the adoption of physical activity policies in elementary schools in Baden-Wuerttemberg, Germany, from the perspective of school principals.

**Methods:**

A cross-sectional study was conducted between May and June 2021. School principals from elementary and special needs schools (*n* = 2,838) were invited to participate in the study. The online questionnaire used was developed based on the CFIR and included questions on school characteristics and constructs of the CFIR domains inner setting, characteristics of individuals, and process. Logistic regression analyses were performed to examine associations between policy adoption and school characteristics as well as CFIR determinants.

**Results:**

In total, 121 schools (4%) participated in the survey, of which 49 (40.5%) reported having adopted a policy to promote physical activity. Positive associations with policy adoption were found for general willingness among teaching staff [odds ratio (OR): 5.37, 95% confidence interval (CI): 1.92–15.05], available resources (OR: 2.15, 95% CI: 1.18–3.91), access to knowledge and information (OR: 2.11, 95% CI: 1.09–4.09), and stakeholder engagement (OR: 3.47, 95% CI: 1.24–9.75).

**Conclusions:**

This study provides a first insight into potential barriers and facilitators at the organizational level of schools that may be relevant to the adoption of physical activity policies, from the perspective of school principals. However, due to a low response rate, the results must be interpreted with caution. A strength of this study includes theoretical foundation through the use of the CFIR. The CFIR could be well-adapted to the school setting and provided valuable support for developing the questionnaire and interpreting the study results.

## Introduction

Physical activity can have a positive impact on children's health and academic achievement (1–3). According to the World Health Organization's (WHO) recommendations on physical activity, children and adolescents aged 5–17 years should do at least an average of 60 min of moderate-to-vigorous intensity of physical activity per day (4). However, the recent Global Matrix 3.0 Physical Activity Report Card analysis showed that only 20–26% of children and adolescents in high- and very high-income countries meet this recommendation (5). To counteract physical inactivity, the WHO recommends different evidence-based policy actions (including school-based concepts) to create active societies, environments, people, and systems (6).

Schools are an important setting for implementing health programs, as they can reach children across various sociodemographic backgrounds and over a relatively long period of time (7–9). Countries such as the United States and Canada have already developed and introduced school-based policies aimed at increasing children's daily physical activity levels (10–12), and the current evidence base underpins the effectiveness of such policies (13, 14). In general, however, the effectiveness of a policy depends not only on the policy itself, but also on the way it is implemented in practice (15).

Implementation can be described as the process of putting to use or integrating innovations within a setting (16). In addition to the setting itself, actors, strategies, the target group, and the characteristics of the policy, may influence this process. In turn, they all interact with an active and dynamic cultural, social, economic, and political context (17, 18). Consequently, the implementation process can be influenced positively (facilitators) and negatively (barriers) in many ways (19).

If organizations such as schools have an intention, make an initial decision or take actions to try or employ an innovation, this is referred to as the implementation outcome “adoption” (20). Adoption occurs at an early to mid-stage of the implementation process and is assessed from the organization's or provider's perspective (20). Either adoption leads to implementation activities or adoption is rescinded (21).

To understand the underlying mechanisms relevant to policy actions, the use of implementation science theories, models, and frameworks can be supportive. Thus, determinant frameworks–in contrast to process and evaluation frameworks–show basically conceptual constructs that can have a potential impact on implementation outcomes (19). One determinant framework that provides a systematic guide for assessing potential barriers and facilitators is the Consolidated Framework for Implementation Research (CFIR) (22). The framework lists 26 key determinants, which are grouped into the following five domains: intervention characteristics, outer setting, inner setting, characteristics of individuals, and process (22).

Previous studies on programs promoting physical activity have examined the processes or underlying determinants of implementation, with some studies focusing on interventions (23, 24) and others on policies (25–28). Compared to interventions, policies are not only individual measures or actions, but provide a framework within which interventions are tendered, developed, financed or implemented (13). Regarding school-based interventions, there is already some evidence on processes and barriers and facilitators that might influence adoption (24, 29–32). In a systematic review by Cassar et al. (24), studies were evaluated for determinants associated with the adoption of school-based physical activity or sedentary behavior interventions. The identified factors were categorized according to Durlak and DuPre's determinant framework (15), whereby most of the facilitators (*n* = 15) and barriers (*n* = 9) related to adoption (e.g., characteristics of the school) could be assigned to the domain “prevention delivery system” (24), which is a domain reflecting organizational capacity. However, research on this topic in relation to policies is rather scarce (11, 29, 33, 34). Furthermore, the research team is not aware of any cross-national, Germany-wide, or south-west Germany-wide studies on barriers and facilitators to the adoption of physical activity policies in elementary schools. So far, there is also little information on what might influence the adoption of a policy from the perspective of school-level decision-makers (11).

Previous studies that have investigated possible determinants to the adoption of physical activity policies in schools have–if at all–used evaluation frameworks [e.g., RE-AIM Framework (35)] which, due to their focus on the evaluation of implementation, are suboptimal for assessing determinants that might impact implementation processes (19). Although numerous frameworks exist for evaluating determinants for policies promoting physical activity (36), we chose the CFIR as it is one of the most widely used frameworks (37, 38), provides a detailed description of constructs (22), and has also been found to be applicable and appropriate for the school setting (39, 40).

The aim of this study, therefore, is to exploratively examine, which barriers and facilitators at the organizational level are associated with the adoption of physical activity policies in elementary schools in Baden-Wuerttemberg (south-west Germany) from the perspective of school principals by using the determinant framework CFIR.

## Materials and methods

### Study design, sample and recruitment

A cross-sectional study of elementary schools and schools for children with special needs was conducted between May and June 2021 in Baden-Wuerttemberg, south-west Germany. In Baden-Wuerttemberg, elementary schools range from first to fourth grade. Without taking early or deferred enrollment and possible repetitions of a grade into account, the age range of students in grades one to four is generally from six to ten years.

**Figure 1:**
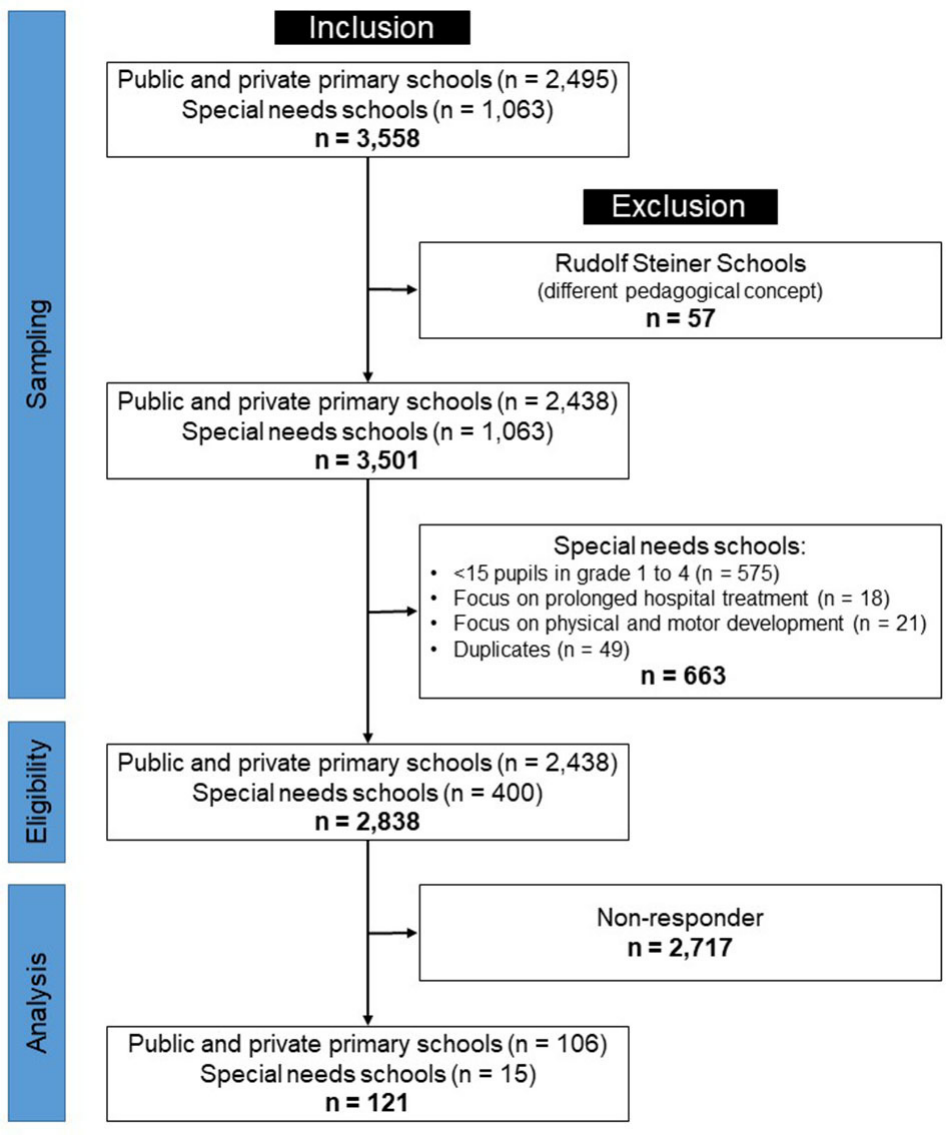
Data sampling flow diagram.

The sample of schools was taken from a database provided by the Federal Statistical Office Baden-Wuerttemberg. All public and private elementary schools (*n* = 2,495) as well as special needs schools (*n* = 1,063) were eligible for participation, with the following exceptions: Rudolf Steiner Schools (*n* = 57) were excluded as they practice a different pedagogical concept. Given that special needs schools usually contain grades five and higher, those with fewer than 15 students in grades one to four were considered to be secondary schools and thus excluded (*n* = 575). Moreover, special needs schools with a focus on students undergoing prolonged hospital treatment (*n* = 18) and physical and motor development (*n* = 21) were excluded, as the promotion of physical activity only plays a subordinate role due to students' physical conditions or is based on special concepts. Furthermore, duplicates among special needs schools (*n* = 49) were removed. Finally, 2,838 schools [elementary schools: *n* = 2,438 (86%); special needs schools: *n* = 400 (14%)] were invited to participate. The data sampling strategy is outlined in Figure 1.

An invitation letter was sent to each school asking all principals and deputy principals to participate in an online survey. The letter contained a brief description of the purpose of the study, all necessary information on the conditions of participation, confidentiality practices, and data protection measures, as well as the contact information of the study office. In addition, the letter included a QR code and URL to access the online questionnaire, as well as an individual study code for each school. To enhance recruitment, two postcards containing key information about study participation and the QR code were enclosed with the invitation letter. In addition, a short video was presented on the front page of the online questionnaire to motivate school principals and provide completion instructions. A reminder letter was sent on June 1, 2021 to increase participation rates. The survey period ran for a total of six and a half weeks between May and June 2021. The study was approved by the ethics committee of Ulm University (Application Number 252/20) as well as the Ministry of Education, Youth and Sports of Baden-Wuerttemberg.

### Questionnaire and measures

The development of the questionnaire is based on the CFIR and existing survey instruments for evaluating physical activity policies or interventions in schools. Individual items of the School Physical Activity Policy Assessment (S-PAPA) (41), School Health Policies and Practices Study (SHPPS) (42), COMPASS school programs and policies (SPP) (43) and “Join the Healthy Boat” (German version) (44) questionnaires were included. The questionnaire was pre-tested by members of the research team, external colleagues, an elementary school principal, and a special education teacher to assess question comprehension, skip patterns, flow and completion time. The final version was transferred to the online survey software Unipark EFS (Enterprise Feedback Suite) (45) and comprised the following sections: (1) personal details, (2) school characteristics, (3) physical activity policies, (4) implementation determinants/attitudes toward policies, (5) physical education, (6) students with disabilities, (7) recess, (8) health promotion, (9) school environment, (10) active way to school, (11) resources and funds, and (12) final questions. The variables used in the present study are described in more detail in the following sections.

### Outcome variable: Policy adoption

Policies can be defined as “*purposeful decisions, plans and actions made by voluntary or authoritative actors in a system designed to create system-level change to directly or indirectly achieve specific societal goals*.” [derived from PEN Consensus with adaptions from Lakerveld et al. (46)]. Based on this definition, policies aiming to promote physical activity at elementary schools in Baden-Wuerttemberg were identified through an internet search and subsequent consultation with the Ministry of Education, Youth and Sports of Baden-Wuerttemberg. Accordingly, there is one mandatory policy (physical education curriculum) and three voluntary policies. The following three voluntary policies, which were focused on, are: (1) National Recommendations for Physical Activity and Physical Activity Promotion, (2) Elementary school with a focus on sport and physical education, (3) Sports and activity-friendly schoolyard.

Through the question “Does your school implement one or more of the following physical activity policies?” and the possible response categories yes/no, the adoption of each individual policy was ascertained. In addition, the participants had the opportunity to name other policies that exist on district/municipal level through an open-ended question. Schools that had reported implementing at least one policy were classified as “adopters,” while schools that had reported not implementing any policies were classified as “non-adopters.”

Respondents who indicated adopting a policy were asked additional questions about the duration of policy adoption, requirements and decision making-process, as well as reasons and requirements for adoption.

### Predictor variables: CFIR determinants

Due to the lack of evidence on potential determinants associated with the adoption of physical activity policies in elementary schools, the selection of CFIR determinants was based on a meta-review that applied the CFIR to examine implementation determinants of school-based physical activity, diet, and sedentary behavior policies (27). In this regard, those CFIR constructs were included in the present study that were indicated as occurring in implementation processes in at least 80% of reviews/stakeholder documents analyzed in the meta-review. Subsequently, the constructs “structural characteristics,” “implementation climate,” “readiness for implementation” (domain inner setting), and “knowledge and beliefs about the intervention” (domain characteristics of individuals) as well as “engaging” (domain process) were included.

To best reflect the CFIR construct structural characteristics, individual items of the aforementioned questionnaires (41–44) were adapted. Participants were asked about the number of students and staff, type of school, care concept, information about the schools surroundings and facilities as well as the minutes of daily recess. Furthermore, the CFIR Interview Guide Tool (https://cfirguide.org/guide/app/#/) was used to formulate questions on CFIR determinants regarding the (sub-)constructs “implementation climate” and “readiness for implementation” as well as on the constructs “knowledge and beliefs about the intervention” and “engaging stakeholders.” A total of 21 questions were asked to reflect the CFIR constructs. All predictor variables, the corresponding questions asked and their respective coding categories are described in Table 1.

**Table 1:** Description of CFIR predictor variables.

**Predictor variable**	**Survey question and response categories**	**Coding categories**
**CFIR domain: Inner setting; Construct: Structural characteristics**		
Number of students^a^	How many children in grades 1 to 4 attend your school?	n/a
Number of students with migration background^a^	How many children at your school have a migration background?	n/a
Numbers of employees^b^	How many personnel in the following positions do you have at your school? *(1) Teachers; (2) Integration assistants; (3) Social education workers; (4) All-day staff*	n/a
Type of school^c^	Please select the school type: *1 = Elementary school, 2 = Special needs school, 3 = Elementary school in combination with a comprehensive school, 4 = Other type of school, 5 = I don't know*	• Elementary school/Elementary and comprehensive school *(ref.)* • Special needs school
Care concept^c^	What is the care concept at your school? *1 = Half-day school; 2 = Open all-day school; 3 = All-day school; 4 = Another care concept; 5 = I don't know*	• Half-day school *(ref.)* • Open all-day school/all-day school
Location of school^d^	What is your school's zip code?	• Rural area *(ref.)* • Urban area
Size of schoolyard^c^	What is the size of the schoolyard at your school? *1 = up to 500 m^2^; 2 = 501*–*1,000 m^2^; 3 = 1,001*–*1,500 m^2^; 4 = 1,501*–*2,000 m^2^; 5 = 2,001 or more m^2^*	• ≤ 1,500 m^2^ *(ref.)* • ≥1,501 m^2^
Number of sport facilities^e^	Which of the following sports facilities are available at your school? *(1) Sports hall; (2) Basketball court; (3) Football pitch; (4) Athletics facility; (5) Swimming pool; (6) None; (7) Other*	• Up to 3 facilities *(ref.)* • 4 or more facilities
Recess minutes^f^	On average, how many total minutes per day does a student receive recess? (during regular instruction time (without afternoon care); recesses when students can be physically active, without breakfast and lunch recesses)	• ≤ 34 min *(ref.)* • ≥ 35 min
**CFIR domain: Inner setting; Construct: Implementation climate**		
General climate^g^	There is a general willingness within the teaching staff to adopt or implement physical activity policies.	n/a
Tension for change^g^	At our school, lack of exercise or physical inactivity among students is a problem.	n/a
Compatibility^g^	The adoption or implementation of a physical activity policy can be well-integrated into existing workflows at our school.	n/a
Relative priority^g^	At our school, health promotion measures already exist (e.g., prevention programs on mental health or nutrition). The adoption or implementation of a physical activity policy tends to take a back seat compared to these.	n/a
Organizational incentives and rewards^g^	Those who are involved in the adoption or implementation of a policy at our school generally receive special recognition for it.	n/a
Goals and feedback^g^	The goals of existing or planned policies are generally clearly formulated and communicated to all persons involved (staff, parents, students).	n/a
Learning climate^g^	At our school, a working climate exists in which principals and/or teachers can express their own fallibility and need for support.	n/a
**CFIR domain: Inner setting; Construct: Readiness for implementation**		
Leadership engagement^g^	At our school, it can be expected that the principal or the person responsible for the project will provide support when introducing or implementing policies.	n/a
Available resources^g^	At our school, we have sufficient resources (time, staff, financial) to adopt or implement PA policies.	n/a
Access to knowledge and information^g^	At our school, we receive or it is planned to receive sufficient information and materials to adopt or implement physical activity policies.	n/a
**CFIR domain: Individual characteristics**		
Knowledge and beliefs about the intervention^g^	Research-based policies cannot be implemented in daily practice.	n/a
**CFIR domain: Implementation process**		
Engaging stakeholders^g^	It is important to involve all possible stakeholders (e.g., teachers, school management, parents, students, researchers, politicians) in the development of policies.	n/a

a The variable was assessed through an open-ended question. For analysis, it was treated as a continuous variable.

b The variables were assessed through an open-ended question. For analysis, responses were summed and treated as a continuous variable.

c The variable was assessed through a single-answer multiple-choice question. For analysis, it was dichotomized.

d Based on the 2002 State Development Plan of the Ministry of Economics Baden-Wuerttemberg, zip codes were assigned to the categories 1 = densely populated areas, 2 = peripheral areas around densely populated areas, 3 = central places and service areas, and 4 = rural areas. 1–3 were categorized as “urban area” and 4 as “rural area.”

e The variables were assessed through closed-ended dichotomous questions (0 = “no”; 1 = “yes”). For analysis, responses were summed and dichotomized based on the median.

f The variable was assessed through an open-ended question. For logistic regression analysis, it was dichotomized based on the median.

g Responses to the corresponding survey question were measured on a five-point Likert scale ranging from 1 = “Do not agree at all” to 5 = “Totally agree.” For the analysis, it was treated as a continuous variable.

### Data analysis

Descriptive statistics such as frequencies and percentages and medians and 25^th^-75^th^ percentiles were computed to summarize categorical and continuous variables, respectively. Normality of data was tested by Kolmogorov-Smirnov test. The chi-square test (categorical variables) and the Mann-Whitney U-test (continuous variables) were used to analyze differences between policy “non-adopters” and “adopters.”

Multiple logistic regression analysis, using the enter method, were performed to examine the association between the outcome variable policy adoption and CFIR determinants on structural characteristics of the school (model 1) and the (sub-) constructs implementation climate, readiness for implementation, knowledge and beliefs about the intervention and engaging stakeholders (model 2). In both models, a complete case analysis (CCA) restricted to schools with no missing values on both the outcome variable policy adoption and predictor variables were performed. Associations are reported as odds ratios (OR) and the respective ninety-five percent confidence intervals (95% CI). A two-sided *p* ≤ 0.05 was considered to be statistically significant. Because of the explorative nature of this study, all results from statistical tests must be interpreted as hypothesis-generating and not as confirmatory. An adjustment for multiple testing was not made. Data were analyzed using IBM SPSS Statistics version 28.0.1.0 (47).

## Results

A total of 121 schools (4% of those eligible) took part in the survey. The questionnaire was completed by 102 principals (84%) and 19 deputy principals. About half of them (56%) had more than 5 years of experience in their position and the majority were women (61%). The distributions of school structural characteristics and the individual CFIR determinants are shown in Tables 2, 3, respectively.

**Table 2:** Descriptive and bivariate analyses of CFIR determinants of the construct structural characteristics with policy adoption (n = 121 schools).

**CFIR inner setting/structural characteristics**	**All (*n* = 121)**	**Policy not adopted (*n* = 72)**	**Policy adopted (*n* = 49)**	**Bivariate test statistic (P-value)**
**Number of students** ^a^				
Median (P25–P75) Min; Max	111 (57–180) 19; 450	86 (45–168) 19; 280	137 (66–181) 30; 450	2,081.50^†^ (0.07)
**Number of students with migration background** ^b^				
Median (P25–P75) Min; Max	17 (8–56) 0; 360	15 (6–55) 0;198	27 (10–62) 1; 360	1,878.00^†^ (0.13)
**Number of employees**				
Median (P25–P75) Min; Max	17 (9–30) 4; 100	17 (9–27) 4; 95	17 (9–34) 4; 100	1,854.50^†^ (0.63)
**Type of school;** ***n*** **(%)**				
Elementary school/elementary and comprehensive school	106 (87.6)	59 (81.9)	47 (95.9)	5.23^‡^^*^
Special needs school	15 (12.4)	13 (18.1)	2 (4.1)	(0.03)
**Care concept;** ***n*** **(%)**				
Half-day school (Open-) All-day school	63 (52.1) 58 (47.9)	40 (55.6) 32 (44.4)	23 (46.9) 26 (53.1)	0.87^‡^ (0.35)
**Location of school;** ***n*** **(%)**				
Rural area Urban area	61 (50.4) 60 (49.6)	37 (51.4) 35 (48.6)	24 (49.0) 25 (51.0)	0.07^‡^ (0.80)
**Size of schoolyard;** ***n*** **(%)**				
≤1,500 m^2^ ≥1,501 m^2^	71 (58.7) 50 (41.3)	48 (66.7) 24 (33.3)	23 (46.9) 26 (53.1)	4.68^‡^ (0.03)
**Sport facilities;** ***n*** **(%)**				
Up to 3 facilities 4 or more facilities	71 (58.7) 50 (41.3)	45 (62.5) 27 (37.5)	26 (53.1) 23 (46.9)	1.07^‡^ (0.30)
**Recess minutes**				
Median (P25–P75) Min; Max	35 (30–40) 20; 100	30 (26–40) 20; 100	35 (30–40) 20; 100	1,973.50^†^ (0.26)

a Missing values n = 6;

b missing value n = 1.

Values are minimum (min), maximum (max), numbers (n) and percentages (%);

† Mann-Whitney-U-test;

‡ Chi-square test;

* Results are based on Fisher's exact test.

**Table 3:** Descriptive and bivariate analyses of CFIR determinants with policy adoption (*n* = 121 schools).

**CFIR item**		**Do not agree at all**	**Do not agree**	**Neither agree nor disagree**	**Agree**	**Totally agree**	**Chi-square test (P-value)^‡^**
		***n*** **(%)**				
**Inner setting/ implementation climate**	General climate	4 (3.3)	4 (3.3)	26 (21.5)	69 (57.0)	18 (14.9)	29.76 (<0.01)
	Tension for change	10 (8.3)	61 (50.4)	9 (7.4)	32 (26.4)	9 (7.4)	2.47 (0.68)
	Compatibility^*^	1 (0.8)	14 (11.7)	38 (31.7)	57 (47.5)	10 (8.3)	5.91 (0.18)
	Relative priority	2 (1.7)	18 (14.9)	33 (27.3)	53 (43.8)	15 (12.4)	0.84 (0.98)
	Incentives and rewards^*^	6 (5.0)	29 (24.2)	52 (43.3)	30 (25.0)	3 (2.5)	5.50 (0.23)
	Goals and feedback^*^	1 (0.8)	14 (11.7)	37 (30.8)	52 (43.3)	16 (13.3)	5.94 (0.17)
	Learning climate^*^	–	1 (0.8)	6 (5.0)	74 (61.7)	39 (32.5)	1.03 (0.91)
**Inner setting/ readiness for implementation**	Leadership engagement^*^	1 (0.8)	1 (0.8)	4 (3.4)	70 (58.8)	43 (36.1)	3.84 (0.43)
	Available resources	15 (12.4)	47 (38.8)	27 (22.3)	28 (23.1)	4 (3.3)	14.52 (<0.01)
	Access to knowledge and information^*^	7 (5.9)	31 (26.1)	43 (36.1)	32 (26.9)	6 (5.0)	26.64 (<0.01)
**Characteristics of individuals**	Knowledge and beliefs about the intervention	5 (4.1)	48 (39.7)	51 (42.1)	17 (14.0)	–	1.56 (0.67)
**Process**	Engaging stakeholder^*^	–	4 (3.3)	14 (11.7)	66 (55.0)	36 (30.0)	6.60 (0.07)

* Due to missing values, the numbers varies slightly. 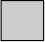 = Median category of five-point Likert scale ranging from 1 = “Do not agree at all” to 5 =” Totally agree”;

‡ bivariate association between policy adoption (non-adopter: n = 72, adopter: n = 49) and predictor variable. Results are based on Fisher-Freeman-Halton exact test.

Overall, 49 schools (40.5% of participating schools) reported implementing one or more physical activity policies at their school. “Elementary school with a focus on sport and physical education” was the policy adopted most frequently (*n* = 38), followed by “Sports and activity-friendly schoolyard” (*n* = 19). In contrast, the “National Recommendations for Physical Activity and Physical Activity Promotion” were adopted by one school. Overall, there were nine schools that implemented two policies. The mean year of policy adoption for “Elementary school with a focus on sport and physical education” and “Sports and activity-friendly schoolyard” was 2011 (earliest: 2000; latest: 2019; missing *n* = 9) and 2007 (earliest: 1995; latest: 2020; missing = 5), respectively.

According to participants, the policies were implemented “completely” at four schools (8%), “mostly” at 27 schools (55%), and “more or less” at nine schools (18%), whereas six participants (12%) indicated that they “don't know” if the policy was implemented as intended (missing: *n* = 3, 6%). One or more of the following reasons were indicated as being crucial for the adoption: decision of school management/principal (*n* = 23, 47%), decision of teaching staff (*n* = 22, 45%), evidence-based policy (*n* = 9, 22%), low costs and high benefits (*n* = 7, 14%), recommendation of another school (*n* = 3, 6%), and recommendation of an authority (*n* = 2, 4%).

Proportionally, the following persons were involved in the decision-making process to adopt a policy: principal (*n* = 42, 86%), teachers (*n* = 40, 82%), deputy principal (*n* = 16, 33%), specialist coordinators, (*n* = 8, 16%), school social workers (*n* = 4, 8%), students' parliament (*n* = 4, 8%), and supervisors (*n* = 3, 6%).

Based on bivariate associations, the data show no differences on school structural characteristics between policy non-adopters and adopters, except for the type of school (*p* = 0.03) and size of schoolyard (*p* = 0.03) (Table 2). Group differences were also found for the CFIR determinants general climate (*p* < 0.01), available resources (*p* < 0.01) and, access to knowledge and information (*p* < 0.01) (Table 3).

For logistic regression analyses, the data of six schools had to be excluded in both models due to incomplete data. Model 1 revealed that there were no significant associations between school structural characteristics and the adoption of a physical activity policy.

Model 2 on the associations to CFIR determinants showed that for each higher level of agreement on the question about the general willingness within the teaching staff, the odds for being an adopter school was increased (OR: 5.37, 95% CI: 1.92–15.05). On the other hand, the determinants tension for change (OR: 0.75, 95% CI: 0.46–1.20), compatibility (OR: 0.77, 95% CI: 0.35–1.71), relative priority (OR: 1.07, 95% CI: 0.60–1.89), organizational incentives and rewards (OR: 1.32, 95% CI: 0.68–2.52), goals and feedback (OR: 1.51, 95% CI: 0.75–3.03), learning climate (OR: 0.70, 95% CI: 0.24–2.10), leadership engagement (OR: 0.35, 95% CI: 0.09–1.34), and knowledge and beliefs about the intervention (OR: 1.47, 95% CI: 0.75–2.88) were not found to be associated with the adoption of a policy. However, each higher level of agreement in terms of available resources (OR: 2.15, 95% CI: 1.18–3.91) as well as receiving sufficient information and materials (OR: 2.11, 95% CI: 1.09–4.09) increased the odds of being an adopter school. In addition, the estimated odds of being an adopter school were increased for each higher level of agreement on the importance of stakeholder involvement in policy development (OR: 3.47, 95% CI: 1.24–9.75). All findings from logistic regression analyses on both models are shown in Table 4.

**Table 4:** Multiple logistic regressions: Model 1 and 2 for school structural characteristics and CFIR determinants as predictors of policy adoption (*n* = 115).

**Predictor variable**	**OR**	**95% CI**	**P-value**
**Model 1 structural characteristics**			
Number of students	1.00	0.99–1.01	0.99
Number of students with migration background	1.00	0.99–1.01	0.78
Number of employees	1.00	0.96–1.04	0.96
Type of School; *n* (%)			
Elementary school/elementary and comprehensive school	ref.		
Special needs school	0.23	0.03–1.64	0.14
Care concept; *n* (%)			
Half-day school	ref.		
(Open-) All-day school	1.26	0.50–3.15	0.63
Location of school; *n* (%)			
Rural area	ref.		
Urban area	1.07	0.46–2.49	0.88
Size of schoolyard; *n* (%)			
≤ 1,500 m^2^	ref.		
≥1,501 m^2^	1.95	0.85–4.44	0.11
Sport facilities; *n* (%)			
Up to 3 facilities	ref.		
4 or more facilities	1.23	0.53–2.85	0.64
Recess			
≤ 34 min	ref.		
≥35 min	1.44	0.63–3.29	0.39
**Model 2 CFIR determinants**			
General climate^a^	5.37	1.92–15.05	<0.01
Tension for change^a^	0.75	0.46–1.20	0.23
Compatibility^a^	0.77	0.35–1.71	0.52
Relative priority^a^	1.07	0.60–1.89	0.83
Organizational incentives and rewards^a^	1.32	0.68–2.53	0.41
Goals and feedback^a^	1.51	0.75–3.03	0.25
Learning climate^a^	0.70	0.24–2.10	0.53
Leadership engagement^a^	0.35	0.09–1.34	0.13
Available resources^a^	2.15	1.18–3.91	0.01
Access to knowledge and information^a^	2.11	1.09–4.09	0.03
Knowledge and beliefs about the intervention^a^	1.47	0.75–2.88	0.26
Engaging stakeholders^a^	3.47	1.24–9.75	0.02

OR, odds ratio; 95% CI, 95% confidence interval; ref., reference category;

a Five-point Likert scale ranging from 1 = “Do not agree at all” to 5 = “Totally agree”.

## Discussion

This study was the first to examine implementation determinants from the inner setting, individual characteristics, and process domain of the CFIR that were associated with the adoption of a physical activity policy in elementary and special needs schools in Baden-Wuerttemberg, Germany. It is striking that the structural conditions of the schools, such as number of students and staff or schoolyard size and number of sports facilities, were not found to be predictors for the adoption of a policy, whereas the general willingness of the teaching staff, available resources, access to knowledge and information, and involvement of stakeholders were significantly associated with the adoption of a physical activity policy.

Based on information provided by participating principals, the present analysis revealed that a large proportion of the teaching staff were generally willing to adopt and implement a policy to promote physical activity. However, the analysis also indicated that the higher the agreement on the level of willingness, the higher the odds of being an adopter school. The question on general willingness asked in the present study, depicted the construct of implementation climate only in a generic way and could not be further explained by the sub-constructs such as learning climate and compatibility. Since organizational climate is a socially-constructed concept that reflects the perceptions of individuals involved in relation to organizational culture (48), it is conceivable that the general willingness within participating schools could have been more accurately described through other factors of the schools' social context (e.g., cultural factors such as values and norms). However, these constructs were not included in the present study.

In our study, only about 30% of participating principals reported having sufficient financial, personnel, and time resources. Furthermore, slightly more than 30% of principals indicated that they receive sufficient information and materials at their school to adopt or implement policies. Both higher levels of agreement on the availability of resources and access to information and materials were positively associated with the adoption of a policy. This finding reflects previous research documenting factors associated with the adoption of school-based physical activity/nutrition policies (29, 33) or interventions (24). Overall, these findings underscore the need for external (financial) support such as from governments.

Another interesting finding of our study is the perceived importance of stakeholder involvement. Thus, 85% of the participating principals indicated that the involvement of stakeholders such as teachers, school management, parents, students, researchers or politicians is important when developing policies. Here, a higher level of agreement was significantly associated with policy adoption. This is consistent with research indicating that stakeholder engagement is the key to successful implementation (49–51). In addition, non-participation or symbolic participation of stakeholders describes a top-down approach (50), which rather hampers successful implementation. Based on responses from the schools that were classified as adopters, our data show that at least 40% of teachers were involved in the decision-making process, which may have facilitated the adoption process. The importance of stakeholder involvement for adoption observed in our study, might be supported by the results of similar research in the school setting (24, 29). However, to better understand the importance of stakeholder engagement on the adoption process of school-based policies, future research should distinguish between individual stakeholder groups.

If we contrast our findings to those from reviews on barriers and facilitators to the processes of implementation of physical activity policies in schools, we can find some overlaps regarding the importance of available resources, access to knowledge and information, and general willingness. Using the Theoretical Domains Framework (52), Nathan et al. (25) and Weatherson et al. (26) identified factors such as “lack of time,” “lack of funds,” “lack of sports facilities,” “lack of training” and “teachers' attitudes toward physical activity (intention)” that may hamper implementation success. Although implementation actions and associated challenges may vary depending on the implementation stage (15, 53), it could be assumed that these factors are of particular importance already during the adoption phase, but also during later implementation stages. In order to make a decision to adopt a policy, time and information might be needed up front (e.g., dealing with the content of the policy, writing applications, applying for funds), and if the school has sufficient staff, this workload could be shared among several people. Furthermore, it could be assumed that the general willingness of all individuals involved supports these processes and individual actions in a positive way. The association with sports facilities observed by Nathan et al. (25) and Weatherson et al. (26), however, were not found in our study. One reason for this could be that the presence of sports facilities is not initially relevant for the adoption from the perspective of school principals. However, as shown by Lounsbery et al. (11), the availability of sports facilities might be of greater importance to teachers in terms of the quality of implementation.

### Strengths and limitations

By applying the CFIR (22) in developing the questionnaire, this study has a solid theoretical foundation. The CFIR can be considered “meta-theoretical” as it was developed by synthesizing constructs from various existing implementation theories (22). It is widely used in implementation science (37, 38) and has already proven to be useful for assessing the implementation of health programs in schools (39, 40). In order to best reflect the selected constructs in a quantitative survey, the CFIR Interview Guide Tool provided sufficient support in formulating corresponding questions. However, whether the respective items actually reflect the constructs adequately is uncertain, as we were not able to conduct validity tests due to limited funding and short study duration. To examine determinants of policy adoption at the organizational level of schools, a variety of constructs from the inner setting, individual characteristics and process domain were included in the analysis. However, possible associations with other domains and their constructs that might better describe, for example, the political and social context in which schools are embedded, were not investigated. Thus, only an incomplete picture could be drawn of the challenges that participating schools faced in adopting a physical activity policy.

Further limitations must be considered when interpreting the present study findings. The overall response rate of schools was low, which limits the generalizability of our results to other schools in Baden-Wuerttemberg. However, the ratio between participating elementary schools and special needs schools was about the same (eligible: 86% and 14%; participated: 88% and 12%). One explanation for the low response rate could be the restrictions imposed to combat the COVID-19 pandemic. Consequently, schools in Baden-Wuerttemberg could still not return to regular operation and principals were facing particular challenges due to organizational overload. Participation in a survey on physical activity among students may therefore not have been a priority. Although measures such as mailing postcards containing the QR-code, a video on the front page of the questionnaire, and a reminder letter were used to increase participation rates, an incentive could not be provided.

It is possible that schools that did not promote physical activity among their students were more likely not to have participated, thus non-response bias may have occurred. As a result, findings may be overestimated in terms of policy adoption. Some questions on the CFIR constructs related to the adoption *or* implementation of policies. Therefore, compared to schools that had not yet adopted a policy, the responses of schools that had already adopted physical activity policies may have been more related to the current situation of implementation rather than the previous circumstances at the time when the policy was adopted. For some schools, the date of implementation was several years ago. Consequently, our study is vulnerable to recall bias. In addition, the complexity of logistic regression models may limit generalizability. Furthermore, no adjustments were made for multiple testing as the research questions were addressed in an exploratory manner.

## Conclusion

The present study provides a first insight into possible barriers and facilitators at school level that might be of importance for decision-makers when adopting physical activity policies. It can be hypothesized that the decision of elementary school principals to adopt a physical activity policy would be facilitated if there is a general willingness within the teaching staff, relevant stakeholders are involved, implementers have access to information and sufficient personnel, financial, and time resources are available. Overall, our experience has been that the CFIR can provide good guidance to assess determinants associated with the adoption of physical activity policies. It could be well-adapted to the school setting and was helpful in designing the study and interpreting the results. Future studies could attempt to explain how the characteristics of the individuals involved affect the adoption of a policy and what importance external influences, such as the political context, may have.

## Data availability statement

The original contributions presented in the study are included in the article/supplementary files, further inquiries can be directed to the corresponding author.

## Ethics statement

The studies involving human participants were reviewed and approved by the Ethics Committee of Ulm University (Application Number 252/20) as well as the Ministry of Education, Youth and Sports of Baden-Wuerttemberg in March 2021. Written informed consent for participation was not required for this study in accordance with the national legislation and the institutional requirements.

## Author contributions

The study was designed by JW, DAS, MFM, and JMS. JW and DAS developed the questionnaire with expert advice from MFM, BM, NL, and JMS. JW conducted the data analysis and interpretation, wrote the draft of the manuscript and integrated author comments, and revisions into the final version. DAS, MFM, AL, NL, and JMS critically revised the subject-specific content of the draft manuscript. BM, SF, AB, and KL contributed substantially to the preparation of the draft manuscript. All authors read and approved the final version of the manuscript.
